# Rice immediately adapts the dynamics of photosynthates translocation to roots in response to changes in soil water environment

**DOI:** 10.3389/fpls.2022.1024144

**Published:** 2023-01-18

**Authors:** Yuta Miyoshi, Fumiyuki Soma, Yong-Gen Yin, Nobuo Suzui, Yusaku Noda, Kazuyuki Enomoto, Yuto Nagao, Mitsutaka Yamaguchi, Naoki Kawachi, Eiji Yoshida, Hideaki Tashima, Taiga Yamaya, Noriyuki Kuya, Shota Teramoto, Yusaku Uga

**Affiliations:** ^1^ Takasaki Advanced Radiation Research Institute, National Institutes for Quantum Science and Technology (QST), Takasaki, Japan; ^2^ Institute of Crop Science, National Agriculture and Food Research Organization (NARO), Tsukuba, Japan; ^3^ Institute for Quantum Medical Science, National Institutes for Quantum Science and Technology (QST), Chiba, Japan

**Keywords:** photosynthate translocation, carbon 11, positron emission tomography, X-ray computational tomograph, positron-emitting tracer imaging system, rice root, intermittent drought stress, root system architecture (RSA)

## Abstract

Rice is susceptible to abiotic stresses such as drought stress. To enhance drought resistance, elucidating the mechanisms by which rice plants adapt to intermittent drought stress that may occur in the field is an important requirement. Roots are directly exposed to changes in the soil water condition, and their responses to these environmental changes are driven by photosynthates. To visualize the distribution of photosynthates in the root system of rice plants under drought stress and recovery from drought stress, we combined X-ray computed tomography (CT) with open type positron emission tomography (OpenPET) and positron-emitting tracer imaging system (PETIS) with ^11^C tracer. The short half-life of ^11^C (20.39 min) allowed us to perform multiple experiments using the same plant, and thus photosynthate translocation was visualized as the same plant was subjected to drought stress and then re-irrigation for recovery. The results revealed that when soil is drier, ^11^C-photosynthates mainly translocated to the seminal roots, likely to promote elongation of the root with the aim of accessing water stored in the lower soil layers. The photosynthates translocation to seminal roots immediately stopped after rewatering then increased significantly in crown roots. We suggest that when rice plant experiencing drought is re-irrigated from the bottom of pot, the destination of ^11^C-photosynthates translocation immediately switches from seminal root to crown roots. We reveal that rice roots are responsive to changes in soil water conditions and that rice plants differentially adapts the dynamics of photosynthates translocation to crown roots and seminal roots depending on soil conditions.

## Introduction

Among the major cereal crops, rice is consumed by more than half of the world’s population, and thus it is important to improve the yield and stable production of rice for food security (Ahmadi et al., 2014). Rice is predominantly produced in irrigated and rainfed lowland paddy systems and consumes more water than other cereals (Maclean et al., 2013). Therefore, rice plants are susceptible to drought stress, which is increasing in incidence and severity worldwide (Kim et al., 2020), adversely affecting rice production (Barnabas et al., 2008). Increasing global temperatures associated with global warming are expected to worsen the drought-related decline in rice yields in future (Lobell and Gourdji, 2012). It is important to understand the response of rice plants to drought stress and, furthermore, to implement breeding programs that lead to yield improvements.

Roots are essential for plants to take up water and nutrients from the soil, affecting plant performance and productivity (Gregory, 2006). Roots are directly exposed to changes in soil condition, and thus they are key organs for elucidating the physiological response of plants to abiotic stress such as drought. Roots can adapt to drought stress as well as efficiently acquire nutrients and water by changing their root system architecture (RSA) and physiological function such as exudation of carbon into the soil, and nutrient uptake using energy (Davies and Bacon, 2003). These changes in RSA and physiological function are driven by photosynthates synthesized in leaves (Li et al., 2010; Hachiya et al., 2014; Yin et al., 2020). To understand in detail the anti-stress strategies of plants under drought stress, it is necessary to investigate the translocation of photosynthates to the roots in addition to changes in RSA.

Three-dimensional (3D) analysis is important to accurately assess the structure and function of roots, which develop intricately in the soil. For the 3D analysis of RSA, magnetic resonance imaging (Jahnke et al., 2009; Metzner et al., 2014), neutron imaging (Tumlinson et al., 2007; Leitner et al., 2014), and X-ray computed tomography (CT) (Jenneson et al., 2003; Zhu et al., 2011; Flavel et al., 2012) have recently been used. However, these methods require considerable time to scan and reconstruct the roots. Moreover, subsequent segmentation of the root is labor intensive. Recently, Teramoto et al. (2020) developed a system that combines high-speed scanning with semi-automated root tracing, thus enabling 3D structural analysis of plant roots with high throughput. To analyze photosynthate translocation dynamics in plants, we developed an imaging technique to visualize and quantify the distribution of ^11^C-labeled photosynthates in plants noninvasively with a spatial resolution of approximately 2 mm by combining (i) a positron-emitting tracer imaging system (PETIS) (Kawachi et al., 2011) and (ii) a small prototype of open-type geometry of positron emission tomography (OpenPET) (Yamaya et al., 2011). ^11^C is a short-lived radioisotope (RI) tracer that emits positrons, with a half-life of 20.39 min. A key advantage of short-lived RI tracers is that *in vivo* measurements can be performed repeatedly using the same plant (Minchin and Thorpe, 2003). We previously completed the PETIS-based investigations on the spatiotemporal distribution of photosynthates in leguminous plants (Kawachi et al., 2011; Yin et al., 2020), *Cannabis sativa* (Kawachi et al., 2006), eggplant (Kikuchi et al., 2008), tomato (Yamazaki et al., 2015; Tsukamoto et al., 2020), and strawberry (Hidaka et al., 2019; Miyoshi et al., 2021a), and OpenPET-based investigations in strawberry fruits (Kurita et al., 2020) and rice roots (Miyoshi et al., 2021b). By combining the high-throughput RSA analysis system developed by Teramoto et al. (2020) with RI imaging system for photosynthates translocation, it would be possible to make detailed analysis of RSA and dynamics of photosynthates translocation to roots in the soil.

Recently, Miyoshi et al., 2021b combined the high-throughput X-ray CT system with the OpenPET to allow rapid acquisition of RSA that developed 3D in the soil and detailed analysis of photosynthate translocation dynamics to rice roots without destroying the plants. Using this system, it was revealed that the activity of photosynthates translocation varied along the individual rice root. The aim of this study is to evaluate the dynamics of photosynthate translocation to rice roots grown in a pot replicating the intermittent drought stresses that can occur in the field, i.e., drought stress and recovery from drought stress due to water influx by using this newly constructed.

## Materials and methods

### Plant material and growth conditions

We used Dro1-NIL with intermediate RSA, which is a rice near-isogenic line developed by introducing the functional allele of *DRO1* gene involved in root depth from Kinandang Patong (Uga et al., 2013). The medium root depth of the DRO1-NIL line selected was suitable for pot imaging by Open-PET. Rice seeds were immersed in water with a fungicide for 24 h at 15°C and pre-germinated in water at 30°C for 2 days. Then, each seed was sown in a plastic pot (diameter of 97 mm, height of 140 mm) filled with Profile^®^ Greens Grade™ (PROFILE Products, Buffalo, Illinois, USA) (Adams et al., 2014) as a soil-like plant growth substrate. Profile is calcined clay, used as a soil suitable for root imaging by X-ray CT because it maintains a constant RSA under both dry and well-watered conditions, and it also retains sufficient water and nutrients for plant growth (Teramoto et al., 2020). The rice plant was grown in a custom-made growth chamber (Nippon Medical & Chemical Instruments, Tennoji-ku, Osaka, Japan) under a photoperiod of 14 h of light and 10 h of dark, with a relative humidity (RH) of 50% during the day and RH of 60% at night (Numajiri et al., 2021). The temperature in the growth chamber was set to increase gradually from 25 to 30°C at the start of the day and to decrease gradually to 25°C toward the end of the day. At night, the temperature was maintained at 25°C. The diurnal temperature program was as follows: zeitgeber time (ZT) 0, 25°C; ZT2, 26°C; ZT3, 27°C; ZT4, 28°C; ZT5, 29°C; ZT6, 30°C; ZT12, 29°C; ZT13, 28°C; ZT14, 27°C; ZT15, 26°C; and ZT16, 25°C. The light condition in the growth chamber varied from 250 to 500 μmol m^-2^ s^-1^ during the day, following a previous report (Teramoto et al., 2020). The diurnal light intensity program was as follows: ZT0, PPFD of 250 μmol m^−2^ s^−1^; ZT1, PPFD of 500 μmol m^−2^ s^−1^; ZT13, PPFD of 250 μmol m^−2^ s^−1^; and ZT14, PPFD of 0 μmol m^−2^ s^−1^.

**Figure 1:**
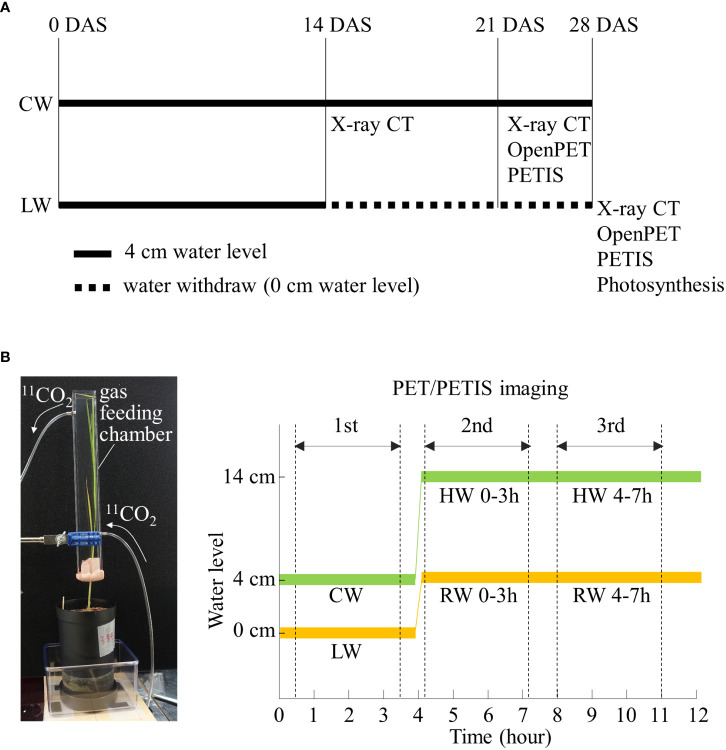
**(A)** Timing and duration of soil water conditions with rice plants grown under control water content (CW) and low water content (LW). The black thick line and black dotted line indicate the cultivation period with water level of 4 cm and 0 cm, respectively. The timing of X-ray CT, OpenPET/PETIS imaging and photosynthesis rate measurements are also shown. **(B)** Photograph of ^11^CO_2_ gas feeding chamber. Time course of water level during first, second, and third OpenPET/PETIS imaging are also shown. RW and HW indicate “recover water content” and “high water content”, respectively. Zero min indicates the start of the lighting period in the growth chamber.

**Figure 1A** shows the timing and duration of different soil water conditions. For the first 14 days after sowing (DAS), Kimura B hydroponic solution (365.0 μM (NH_4_)_2_SO_4_, 91.0 μM K_2_SO_4_, 547.0 μM MgSO_4_, 183.0 μM KNO_3_, 365.0 μM Ca(NO_3_)_2_, 182.0 μM KH_2_PO_4_, 4 mg/L FeC_6_H_5_O_7_/nH_2_O, pH 5.5) (Yoshida, 1976) was supplied to the plastic pot to a height of 4 cm from the bottom of the pot. The water level of 4 cm allowed water to be evenly distributed in soil and was an optimized balance to prevent soil being either flooded or dry. This optimal soil water content level is referred to as “control water content” (CW). After 14 DAS, hydroponic solution was withdrawn for soil to dry until 28 DAS. During this period, plants showed symptoms of response to drought, i.e., leaf rolling and slower growth. This drier soil water content is referred to as “low water content” (LW). CW plants at 28 DAS had significantly higher shoot and root biomass compared to LW plants at the same stage (**Supplementary Figure S1**). CW plants at 23 DAS with similar root and shoot biomass compared to LW plants 28 DAS were chosen as control plants to allow for comparison of photosynthates translocation in similar root biomass.

### X-ray CT system

The underground part of rice plant was scanned using the X-ray CT system inspeXio SMX-225CT FPD HR (Shimadzu Corporation, Nakagyo-ku, Kyoto, Japan), as previously reported (Teramoto et al., 2020), on the day before OpenPET or PETIS experiments. Tube voltages of 225 kV and tube currents of 500 μA were used. The X-ray source was positioned at a distance of 366 mm from the test plant and 800 mm from the detector. To harden the X-ray beams, 1.0 mm copper filter was used. Each CT scan took 10 min. The matrix size and voxel size of the reconstructed images were 1024 × 1024 × 788 and 0.18 mm × 0.18 mm × 0.18 mm, respectively. The root segments in the X-ray CT images were visualized using a combination of median filtering and edge detection algorithms, in accordance with the RSAvis3D method (Teramoto et al., 2020). Furthermore, the RSA was vectorized using a tracking algorithm, and the center of gravity of the root voxels was used to determine the tracking direction in accordance with the RSAtrace3D method (Teramoto et al., 2021).

### ^11^CO_2_ tracer production

^11^CO_2_ was produced by the ^14^N(p,α)^11^C reaction induced by bombarding pure nitrogen gas with 10 MeV protons from an AVF cyclotron located at Takasaki Ion Accelerators for Advanced Radiation Application (TIARA), QST, Japan (Ishioka et al., 1999). The irradiated gas containing nitrogen gas and ^11^CO_2_ was passed through a stainless steel trap (^11^CO_2_ trap) immersed in liquid nitrogen, and only the ^11^CO_2_ gas was collected as dry ice in the trap. In this study, approximately 35 MBq of ^11^CO_2_ was collected and transferred to the OpenPET and PETIS imaging experiments.

### OpenPET and PETIS imaging

**Figure 1B** shows the protocol of OpenPET/PETIS imaging. All the leaves of the rice plant were inserted into an acrylic box (gas feeding chamber) with an inside dimension of 20 mm length, 30 mm width, and 300 mm height. The gas feeding chamber was sealed at the petiole with resin clay (Tak Model Bloc; Tak Systems Corporation, Osaka, Japan) to prevent leakage of the fed ^11^CO_2_. An air pump was connected to the inlet of the chamber and ambient air was pumped into the chamber at a constant rate of 500 mL min^-1^. A ^11^CO_2_ trap was connected in the middle of the air flow path between the air pump and the gas feeding chamber. ^11^CO_2_ was pushed out of the ^11^CO_2_ trap into the chamber to feed leaves. After 5 min of ^11^CO_2_ feeding, the ^11^CO_2_ trap was disconnected from the air flow path. ^11^CO_2_ passed through the chamber within 1 min. The unassimilated ^11^CO_2_ by the leaves was collected in soda lime (Soda lime No. 1; Wako Pure Chemical Industries, Ltd., Osaka, Japan) in an acrylic tube connected to the outlet of the chamber. The radioactivity of the soda lime was quantified with a curie meter 10 min after ^11^CO_2_ feeding to estimate the amount of ^11^C fixed by the plant in each imaging experiment. Then, the plastic pot was set in the middle of field of view (FOV) (diameter 110 mm, length 145 mm) of the vertically placed OpenPET. The OpenPET has been demonstrated to visualize and assess the dynamics of photosynthates translocation to rice roots which develop 3D throughout the FOV without loss of spatial resolution (Miyoshi et al., 2021b). The OpenPET measurements, lasting 170 min, then started, and ^11^C distribution images were acquired. The environmental conditions around the plant were set to 500 µmol m^-2^ s^-1^ of light intensity and 30°C of air temperature during imaging experiments. OpenPET data were reconstructed every 5 min using the ordered subset expectation maximization (OS–EM) method. The matrix size of the reconstructed image was 76 × 76 × 84 and the voxel size was 1.5 × 1.5 × 1.5 mm. The reconstructed OpenPET images were corrected for the radioactive decay of ^11^C (half-life = 20.39 min).

Partitioning of ^11^C-labeled photosynthates in rice roots under LW and CW were imaged by OpenPET. Furthermore, the translocation of photosynthates to rice roots in response to changes in the soil water condition from irrigation under LW was examined. The OpenPET imaging was repeated three times for each plant sample. After the first OpenPET imaging, the plant sample was irrigated up to 4 cm from the bottom of the pot. After 10 min of irrigation, ^11^CO_2_ was fed to the leaves and OpenPET imaging was performed for the second time. After the second OpenPET imaging, i.e., 4 hours after irrigation, ^11^CO_2_ was fed to the leaves and OpenPET imaging was carried out for the third time. The first, second, and third OpenPET imaging were defined as LW, “recover water content 0-3 h (RW 0-3 h)” and “recover water content 4-7 h (RW 4-7 h)”, respectively (**Figure 1B**). RW 0-3 h refers to imaging experiments conducted from 10 min to 3 hours after the start of irrigation. By using software RSAadjust3D (https://github.com/st707311g/RSAadjust3D ) to adjust the position of PET and CT images, the obtained OpenPET images were rescaled and coordinated with RSA images obtained from RSAtrace3D. Then, the rescaled and coordinated OpenPET images and RSA images were superimposed using the open-source software OsiriX (Rosset et al., 2004).

PETIS was installed in a plant growth chamber so that the ambient environmental conditions could be controlled during the experiments. Although PET imaging analysis is performed in a three-dimensional plane, a wider area can be visualized by using this system. In this study, the plastic pot with roots was positioned in the PETIS FOV, which was 119.9 mm wide and 187.0 mm high. All rice leaves were inserted into the gas feeding chamber. Approximately 35 MBq of ^11^CO_2_ was administered to the leaves, as in the OpenPET imaging protocol. PETIS imaging started as soon as ^11^CO_2_ was injected, and PETIS images were acquired every 10 s for 180 min.

PETIS imaging was used to analyze the partitioning pattern of ^11^C-labeled photosynthates to roots in response to re-irrigation under LW using the same protocol as the OpenPET study. Furthermore, the translocation of photosynthates to roots in response to changes in the soil water condition by flooding rice plants near the soil surface under CW was analyzed. After PETIS imaging of photosynthate translocation to roots under CW, irrigation was applied from the bottom of the plastic pot to the surface of the soil. After 10 min of flooding, ^11^CO_2_ was fed to the leaves and PETIS imaging was performed for the second time. After the second PETIS imaging, i.e., 4 hours after the flooding, ^11^CO_2_ was fed to the leaves and PETIS imaging was carried out for the third time. The first, second, and third PETIS imaging were named CW, “high water content 0-3h (HW 0-3 h)” and “high water content 4-7h (HW 4-7 h)”, respectively (**Figure 1B**).

### OpenPET/PETIS image data analysis

The translocation of ^11^C to rice roots was analyzed by setting the regions of interest (ROIs) around the seminal roots and crown roots in the OpenPET images. Time-course analyses of ^11^C radioactivity within each ROI involved the generation of time-activity curves (TACs) from the signal intensities (counts per second; cps) obtained using AMIDE (Loening and Gambhir, 2003) and Image J (version 1.50) (National Institutes of Health, Bethesda, MD, USA; http://rsb.info.nih.gov/ij/ ). All cps values were corrected for the radioactive decay of ^11^C and normalized by the amount of ^11^C fixed by the plant in each imaging experiment (i.e., cps/MBq).

### Photosynthesis rate during imaging

Time-course changes in the photosynthesis rate under LW, RW 0-3 h and RW 4-7 h during PETIS imaging were analyzed to assess the process of recovery from drought stress of rice plant. CO_2_ concentration sensors (GMP252, Vaisala Oyj, Helsinki, Finland) were attached to the air flow paths at the inlet and outlet of the gas feeding chamber, and the photosynthesis rate of rice leaves in the chamber during PETIS imaging was calculated using the following equation.


$$Pn=\;\frac{v}{22.4}\left({\left[CO_{2}\right]}_{\text{in}}-{\left[CO_{2}\right]}_{\text{out}}\right)$$

Here *Pn* is the net photosynthesis rate [μmol plant^-1^ s^-1^], *v* is the airflow rate passing through the chamber [L s^-1^], [*CO*_2_]_in_ is the CO_2_ concentration of inflow air [μmol mol^-1^], [*CO*_2_]_out_ is the CO_2_ concentration of outflow air [μmol mol^-1^]. The constant value of 22.4 corresponds the volume of 1 mol of air [L mol^-1^].

## Results

### Root system architecture in soil

**Figure 2:**
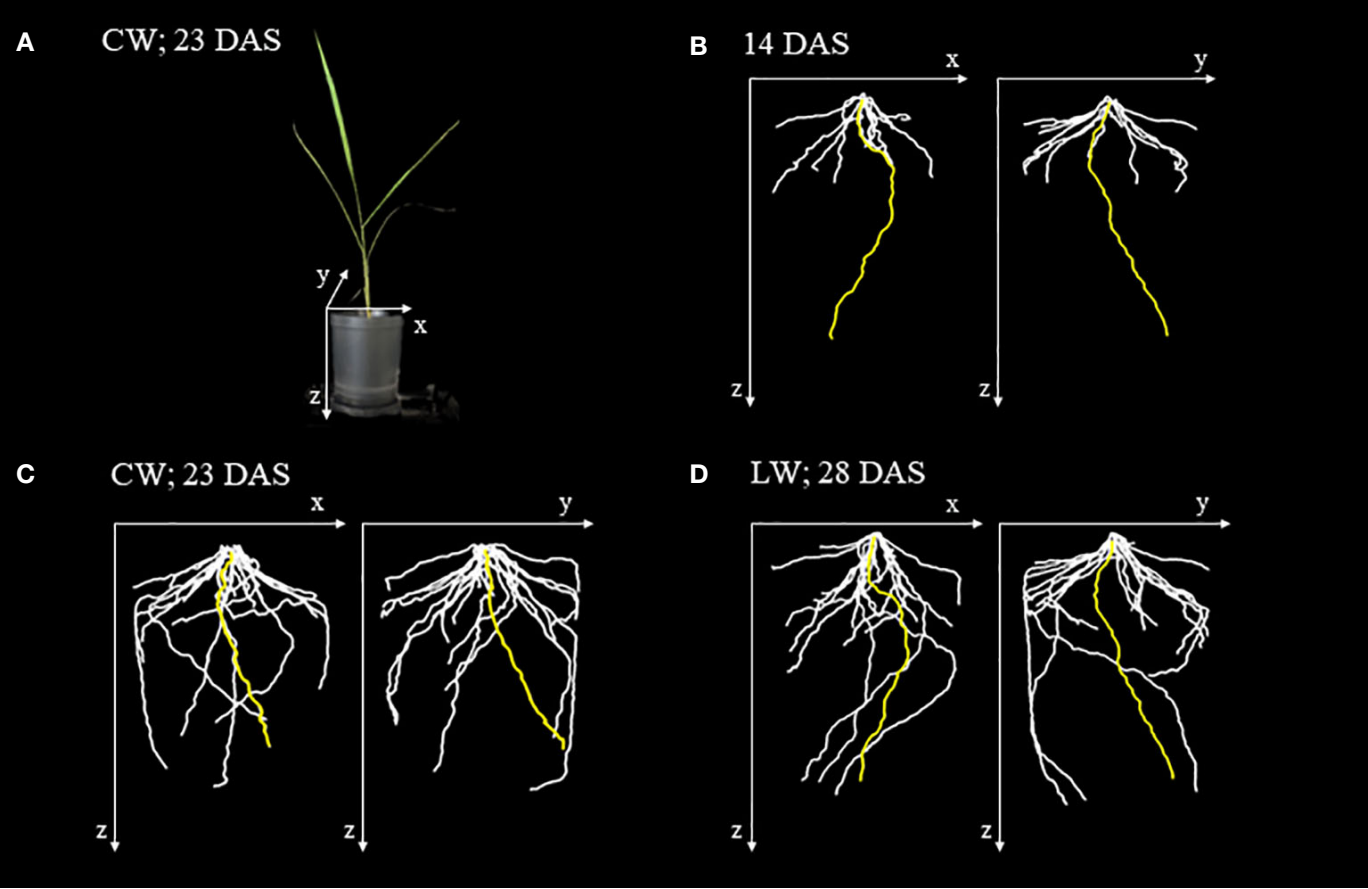
X-ray images of the root system architecture (RSA) of rice plant grown in a plastic pot. **(A)** Images of rice plant grown in a plastic pot 23 days after sowing (DAS) under control water content (CW). **(B)** RSA of rice plant 14 DAS based on 3D reconstructed image obtained from X-ray CT image. The yellow-, white-colored lines indicate seminal and crown roots, respectively. The x, y, and z axes correspond to the axes shown in **(A)**. **(C)** RSA of the rice plant under the CW at 23 DAS. **(D)** RSA of the rice plant under the low water content (LW) at 28 DAS. This is the same plant shown in **(B)**.

X-ray CT imaging visualized the structure of seminal root growing straight down from the shoot base of the rice plant and crown roots extending diagonally downward from the base (**Figure 2**). At 14 DAS, seminal roots were longer than crown roots and extended near the bottom of the pot (**Figure 2B**). At 23 DAS and 28 DAS, the crown roots continued to elongate, extending along the wall to the bottom of the pot while the seminal roots slowed down to elongate under both CW and LW (**Figures 2C, D**).

### Photosynthesis rate of rice leaves during imaging experiments

**Figure 3:**
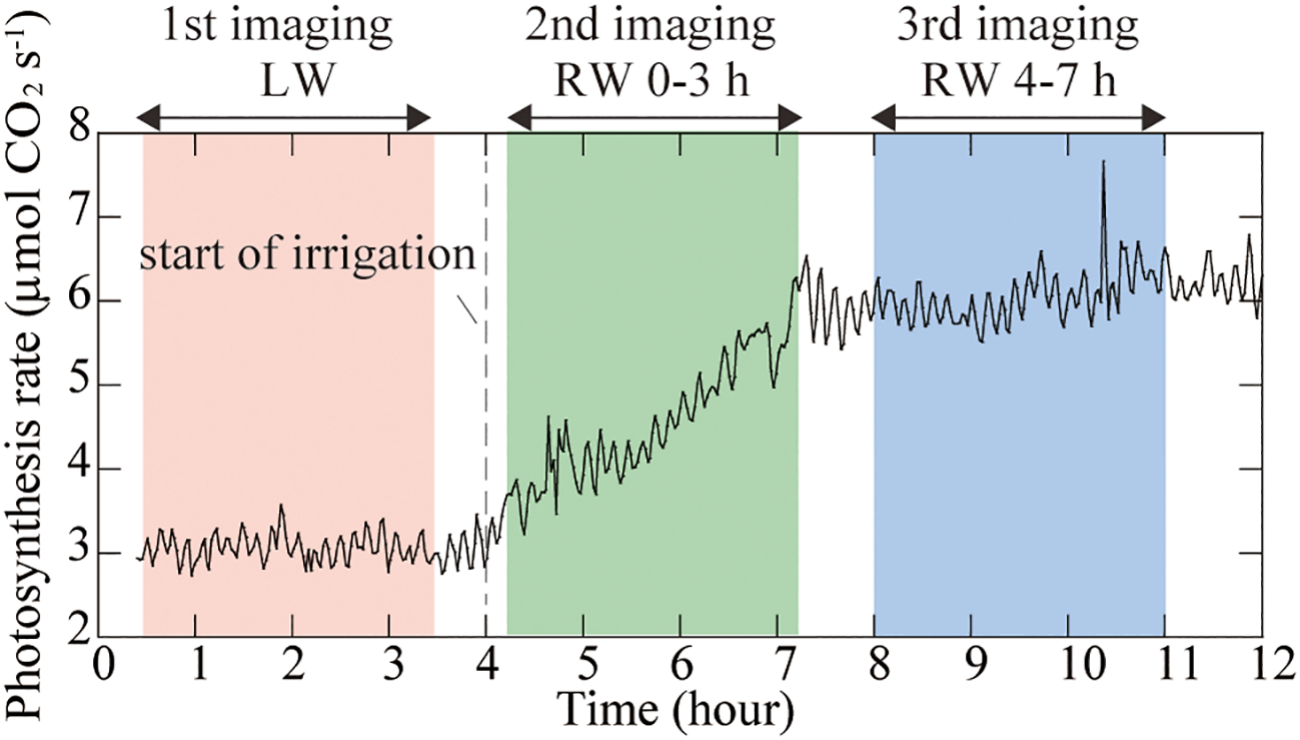
Time course of the photosynthesis rate of rice leaves during PETIS imaging. Zero hour indicates the start of the lighting period. The light pink area indicates the first PETIS imaging under the low water content (LW), the light green area indicates the second PETIS imaging under the recover water content 0-3 h (RW 0-3 h), and the light blue area indicates the third PETIS imaging under the recover water content 4-7 h (RW 4-7 h). Irrigation started 4 h after the start of the light period.

During PETIS imaging under LW, the photosynthesis rate of rice leaves remained at approximately 3 µmol CO_2_ s^-1^ (**Figure 3**). During the second imaging under the RW 0-3 h, the photosynthesis rate was approximately 3 µmol CO_2_ s^-1^ immediately after the start of PETIS imaging and increased to 6 µmol CO_2_ s^-1^ by the end of imaging, 3h later. During the third imaging under the RW 4-7 h, photosynthesis rate remained at approximately 6 µmol CO_2_ s^-1^.

### Visualization of ^11^C-labeled photosynthates translocation to roots

**Figure 4:**
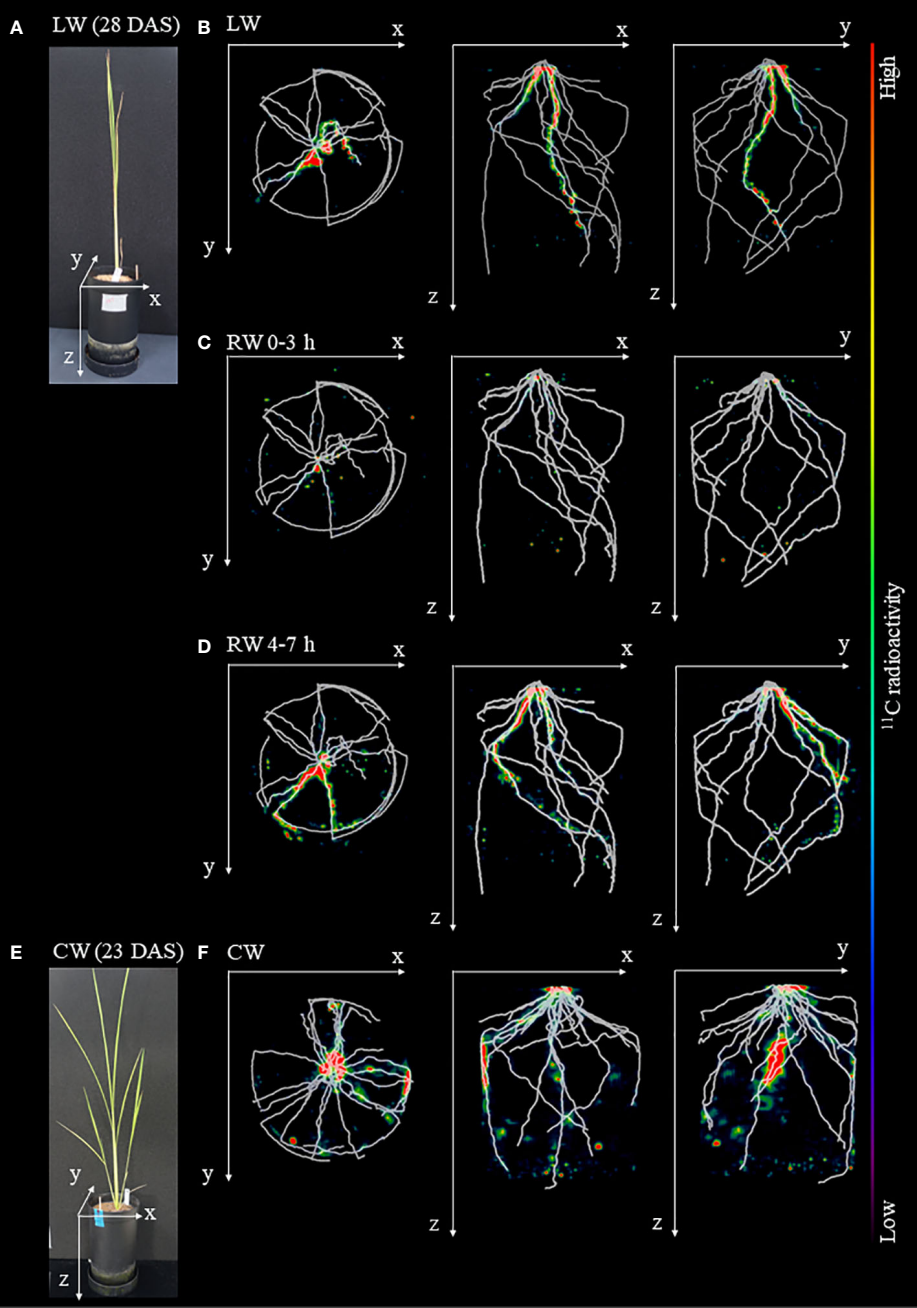
Fused images obtained from OpenPET imaging experiments and X-ray CT. **(A)** Photograph of rice plants under drought condition before the start of OpenPET imaging at 28 DAS. Fused images of the distribution of ^11^C-photosynthates obtained from OpenPET imaging and root system architecture (RSA) of plants growing under in **(A)** obtained from X-ray CT under **(B)** low water content (LW), **(C)** recover water content 0-3 h (RW 0-3 h), and **(D)** recover water content 4-7 h (RW 4-7 h), viewed from the top of the pot (xy plane), front of the pot (xz plane), and side of the pot (yz plane). Images obtained from OpenPET imaging experiments under the control water content (CW) are also shown. **(E)** Photograph of rice plants under CW at 23 DAS. **(F)** Fused images obtained from OpenPET and X-ray CT under the CW, viewed from the xy plane, xz plane, and yz plane. The monochrome images show the RSA, and the color images show the distribution of ^11^C-photosynthates.

The OpenPET-CT imaging was used to visualize in three dimensions the translocation of ^11^C-labeled photosynthates from rice leaves to roots in the soil (**Figure 4** and **Supplementary Figure S2**). The obtained OpenPET-CT images showed that ^11^C-photosynthate partitioning was quickly responsive to changes in soil water conditions. ^11^C-photosynthates actively translocated to the seminal roots and crown root under LW (**Figure 4B**). However, under the RW 0-3 h, ^11^C-photosynthates translocation to seminal root was barely confirmed. Instead, they translocated to some crown roots (**Figure 4C**). Under the RW 4-7 h condition, translocation of ^11^C-photosynthates to crown roots was enhanced (**Figure 4D**). Under the CW, ^11^C-photosynthate translocation to the seminal roots was barely observed, while the active translocation to crown roots was observed (**Figure 4F**). ^11^C-photosynthates were translocated unevenly to some crown roots, as observed under the RW 0-3 h and RW 4-7 h.

**Figure 5:**
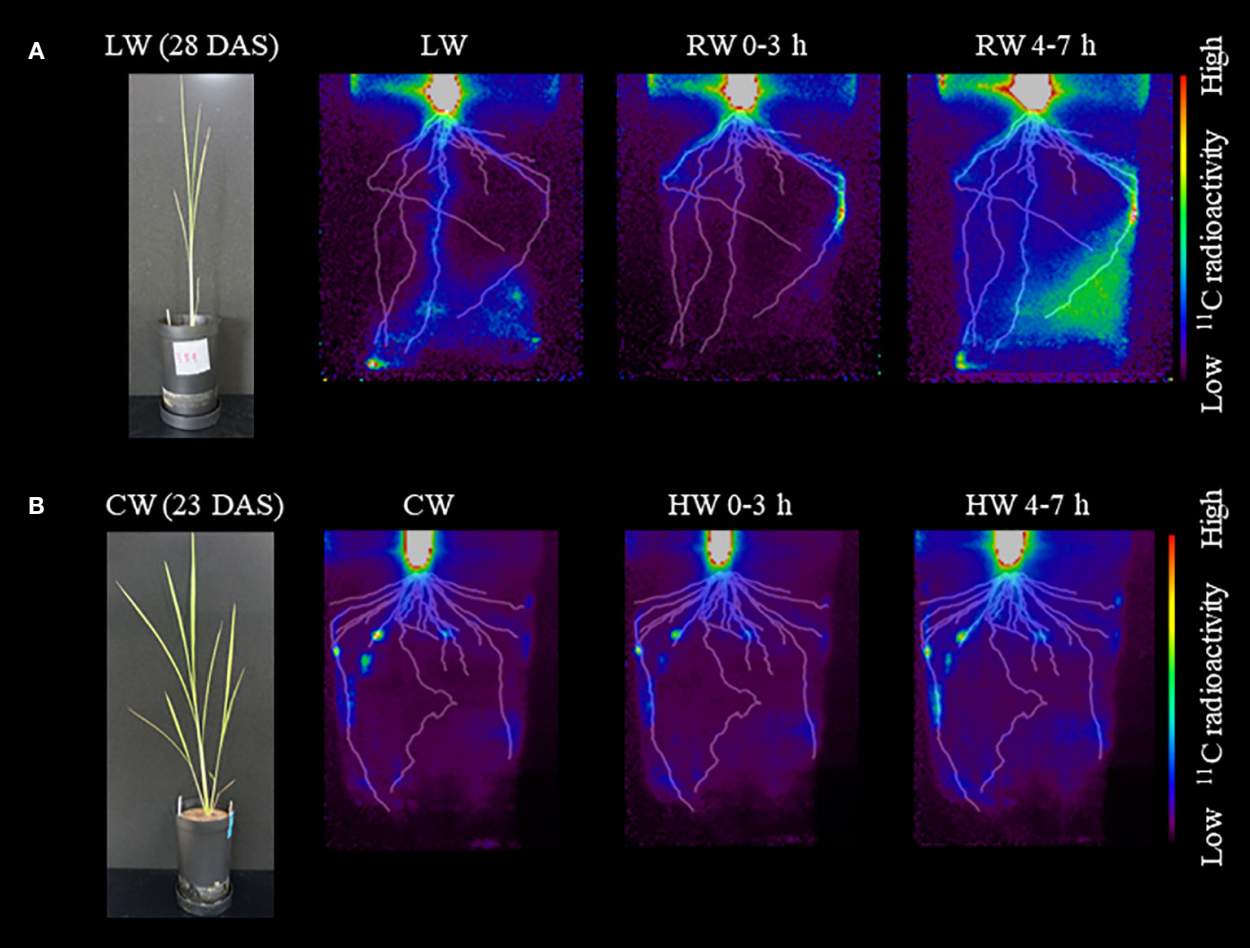
Typical images obtained from PETIS imaging experiments under **(A)** low water content (LW), recover water content 0-3 h (RW 0-3 h), and recover water content 4-7 h (RW 4-7 h). Photograph of rice plant before the start of PETIS imaging at 28 DAS is also shown. PETIS images under **(B)** control water content (CW), high water content 0-3 h (HW 0-3 h), and high water content 4-7 h (HW 4-7 h). Photograph of rice plant at 23 DAS is also shown. Fused images show the distribution of ^11^C-photosynthates obtained from PETIS imaging and the root system architecture (RSA) obtained from X-ray CT under each treatment viewed from the front of the pot. The monochrome images show the RSA, and the color images show the distribution of ^11^C-photosynthates.

PETIS imaging showed similar changes in ^11^C-photosynthate translocation in response to soil water conditions (**Figure 5A**). Under the LW, translocation of ^11^C-photosynthates to the root extending directly down from the base, i.e., the seminar root, was active. Under the RW 0-3 h, translocation of ^11^C-photosynthates to seminal roots did not occur, while translocation of ^11^C-photosynthates to roots extending obliquely downward from the base, i.e., crown roots, became active. Under the RW 4-7 h condition, translocation of ^11^C-photosynthates to these crown roots was enhanced. There was also increased ^11^C-photosynthates translocation to the roots, which was not observed in RW 0-3 h. Furthermore, PETIS imaging under the CW followed by plant flooding confirmed that this change in soil water environment did not significantly change the patterns of ^11^C-photosynthates translocation to the roots (**Figure 5B**). Under the CW, ^11^C-photosynthate translocation to some crown roots was active, similar to results of OpenPET imaging. Under the HW 0-3 h and HW 4-7 h, translocation to these crown roots was also active, while no translocation to other roots was observed. The experiments shown in **Figure 5** were performed on different plant and the same translocation trend was observed (**Supplementary Figure S3**).

### Quantitative analysis of ^11^C-photosynthates translocation to roots

**Figure 6:**
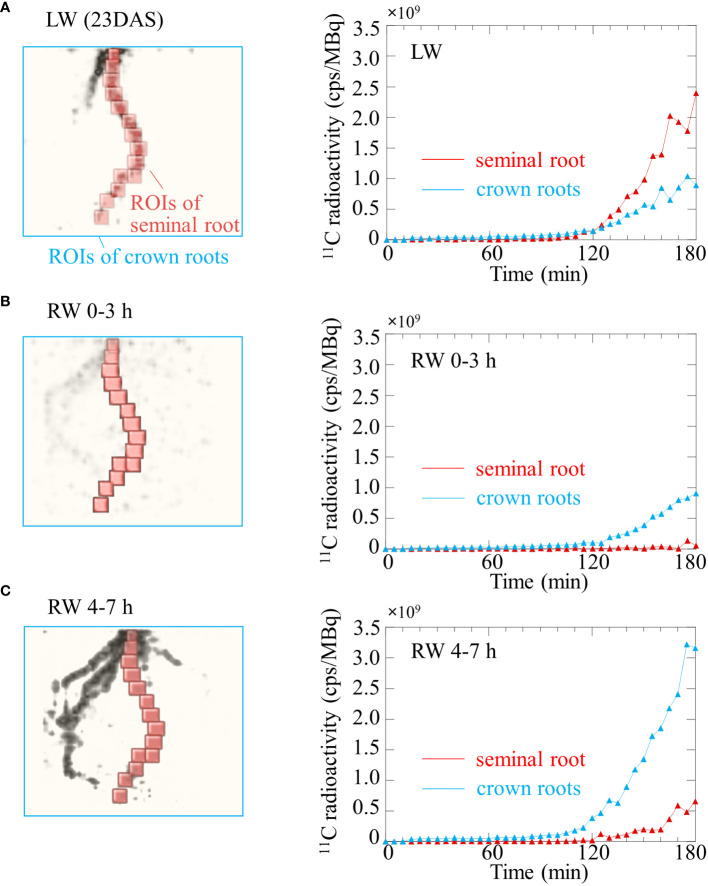
Time course of ^11^C radioactivity in the seminal root (red line) and crown roots (blue line) of rice plant using OpenPET experiments shown in Figure 4 under **(A)** low water content (LW), **(B)** recover water content 0-3 h (RW 0-3 h), and **(C)** recover water content 4-7 h (RW 4-7 h). Regions of interest (ROIs) of the primary root and crown roots placed on OpenPET images are also shown. ROIs of crown roots does not include the seminal root.

The TAC obtained from OpenPET imaging under the LW revealed that ^11^C-photosynthates translocation to seminal and crown roots began approximately 110 min after ^11^CO_2_ was fed to the leaves (**Figure 6A**). The translocation rate of ^11^C-photosynthates was faster in seminal roots than in crown roots. The normalized radioactivity of ^11^C-photosynthates at the end of OpenPET imaging was approximately 2.4 cps/MBq in the seminal roots and 0.9 cps/MBq in the crown roots (**Figure 6A**). Under the RW 0-3 h, translocation to the seminal roots was not confirmed in the TAC of **Figure 6B**. Translocation to the crown roots started approximately 130 min after the injection of ^11^CO_2_. The normalized radioactivity of ^11^C-photosynthates translocated throughout the crown roots during OpenPET imaging was approximately 0.9 cps/MBq (**Figure 6B**). Under the RW 4-7 h, ^11^C-photosuntahte translocation to crown roots was active. Translocation to the seminal roots was again detected in the TAC of **Figure 6C**. Translocation to the crown and seminal roots started approximately 105 min and 125 min after the injection of ^11^CO_2_, respectively. The normalized radioactivity of ^11^C-photosynthate in the seminal and crown roots at the end of the OpenPET imaging was approximately 0.6 cps/MBq and 3.2 cps/MBq, respectively (**Figure 6C**). The total normalized ^11^C radioactivity of seminal and crown roots was similar in LW and RW 4-7h (3.3 and 3.8 cps/MBq, respectively).

## Discussion

Using the advantage provided by the short half-life of ^11^C (20.39 min), which allowed repeated experiments to be performed on the same plant, we visualized the translocation of photosynthates to roots during recovery from drought stress in the same plant using ^11^C tracer with OpenPET and X-ray CT. The OpenPET results were analyzed with reference to the RSA obtained from X-ray CT, revealing that photosynthates were unevenly translocated to some roots (**Figure 4**). This unevenness may be caused by differences in the ability of each root to demand photosynthate, i.e., sink strength (Chamont, 1993). Under the LW, the photosynthates translocation to seminal root was active, while under the RW, the destination of photosynthates translocation immediately switched from seminal root to crown roots. To the best of our knowledge, this is the first study to discuss the response of each root to drought stress and its recovery from drought by sorting roots by structural characteristics, such as seminal root and crown roots.

We found that photosynthate translocation to seminal roots extending from the base of the rice plant to the deep layers of soil became active under drought stress. Although some crown roots developed along the pot wall and reached the same depth as the seminal roots (**Figure 2D**), photosynthate translocation to these crown roots did not occur under drought stress. These results imply that seminal roots and crown roots at different growth stages have the different capacity to acquire and retain water, even though the depth of each root is the same under drought stress. When the roots were removed from the pot after OpenPET/PETIS imaging was completed, more lateral roots were found developing from the seminal roots than from the crown roots (personal observation). It has been reported that the plants adapt to heterogeneous water conditions by branching their lateral roots at water contact points (Bao et al., 2014; Robbins and Dinneny, 2018). Lateral roots contribute to increased water absorption and facilitate the extraction of nutrients essential for plant growth and development (Santos Teixeira and ten Tusscher, 2019). For this reason, we suggest that the seminal roots had a high-water absorption efficiency in our pot condition under the LW. Further physiological experiments are required to verify this implication, such as observation of root forms with active photosynthate translocation depending on the water condition in soil, or cutting seminal roots and evaluate the translocation dynamics.

In rice plants that recovered from the LW by re-irrigation, the sink strength of each root was changed. The ^11^C-photosynthate translocation to crown roots were more active than to seminal root (**Figures 4B, C**, **5A**). In contrast, in rice plants flooded to the soil surface following the CW, the sink of photosynthates did not change (**Figure 5B**). These results indicate that the switching of the photosynthate sink in the roots of rice plants is triggered by a specific environmental change, i.e., water added to dry soil. These results imply that, under drought stress, photosynthates translocate to the lower roots of rice plants to promote elongation and thus absorption of water in the deeper roots layers of soil. When more water is available, rice plants may switch the destination of photosynthate translocation to the entire crown root, resulting in absorption of more water and soluble nutrients from a wider area of the soil. This switching of the photosynthate sink was observed under RW 0-3 h (**Figure 6B**), suggesting that switching is a rapid response to the addition of water. We suggest that rice adapts to changes in the soil water conditions by switching the roots to which photosynthates are partitioned. The phenomenon of switching photosynthate translocation in a short period of time without changing the source leaves, as in the present study, has not been reported. To clarify the mechanism of the switch in photosynthate translocation to the roots due to the soil water conditions, it is required to analyze gene expression related to the translocation, such as sucrose transporters, expressed in the roots and stem base of the rice plant, and to evaluate translocation dynamics when water is only supplied to crown roots, not seminal roots. Recently, it was reported that sucrose transporters SWEET11 and SWEET12 were phosphorylated in response to drought stress (Chen et al., 2022). The phosphorylation enhanced sucrose transport activity which results in elevated sucrose contents in root and improved root growth under drought stress conditions (Chen et al., 2022). These transporters might be involved in the regulation mechanism of the switch in photosynthate translocation to the roots.

After addition of water on dry soil, the photosynthesis rate of rice leaves recovered immediately (**Figure 3**), whereas translocation of photosynthates was not immediate and was detected 120 min after irrigation (**Figure 6B**). To mitigate drought stress, plants reduce transpiration and photosynthesis rate by closing stomata (Yokoyama et al., 2021). The photosynthesis rate of leaves under drought stress increased simultaneously with irrigation, indicating that the plant responded immediately to the soil water status by opening the leaf stomata. When the photosynthesis rate was still increasing, ^11^C radioactivity was observed in crown roots but not seminal roots, suggesting that photosynthates translocation to seminal roots was suppressed (**Figure 6B**). When photosynthesis rate reached a constant value, the amount of photosynthates translocated to the seminal root and crown roots increased (**Figure 6C**). Miyoshi et al., 2021a and Nakai et al., 2022 suggested that under conditions in which photosynthates are not sufficiently stored in the leaf, the loading of photosynthates from the leaf to phloem is inhibited. The obtained results from present study suggest that at the early recovery from drought stress did not affect photosynthates translocation to crown roots but suppressed translocation to seminal roots, and subsequently, when leaf photosynthesis stabilized at increased rates, the photosynthates translocation to roots increased in general. In addition, total radioactivity of ^11^C-photosynthates translocated to seminal and crown roots were almost the same in LW and RW, even though the photosynthesis rate in LW was about half that in RW, suggesting that sink strength in the belowground part increases under drought conditions compared to well-watered conditions.

In this study, under pot condition with drought stress, photosynthate translocation to the seminal roots extending deep into the soil became active (**Figures 4B**, **5A**). Using the rootbox-pinboard method (Kono et al., 1987), which allows two-dimensional observation of RSA, it was suggested that rice cultivars with high tolerance to soil water fluctuations, such as drought stress after waterlogging conditions and subsequent re-irrigation, efficiently utilized the photosynthates partitioned from leaves depending on the soil water conditions (Suralta and Yamauchi, 2008; Wang et al., 2009). It was also reported that lateral root development was significantly enhanced in the deeper layers under drought stress (Galamay et al., 1992). Root phenotypic plasticity was suggested to play an important role in rice growth under changing soil changing soil water conditions (Banoc et al., 2000), however, the mechanism by which the RSA is altered by the soil water conditions remained unclear. This study indicated that drought stress alters root development by modifying the photosynthates translocation to roots, which was not revealed by morphometric analysis using rootbox-pinboard method. Henry et al. (2011) reported that a deep root system enables rice plants growing in water-limited conditions to absorb water from the soil. It was also reported that under drought stress, allocation of photosynthates to the root is prioritized to promote root growth to the deep layers of soil, which contains sufficient water content, thus increasing the surface area of the root for water absorption (Eapen et al., 2005; Ryser, 2006; Hammond and White, 2008; Dietrich et al., 2017; Chen et al., 2022). This study suggests that the changes in RSA reported by previous studies are also triggered by changes in translocation patterns in response to soil water conditions. It also indicates that the differences among rice cultivars in root phenotypic plasticity that adapts to changes in the soil water conditions in the previous study (Banoc et al., 2000) may be due to differences in the ability of photosynthates translocation to roots found in this study.

## Conclusions

We successfully visualized the translocation of photosynthates in rice plants non-destructively as the plants recovered from drought stress, using ^11^C tracers with OpenPET and X-ray CT. We found that under drought stress, photosynthates translocated to the seminal roots, and when water was added to the dry soil, the photosynthate sink immediately switched from the seminal root to the crown roots. The results of this study indicates that a comprehensive analysis of not only the aboveground parts of plants, but also the underground parts is important to understand plant responses to drought stress. The combined OpenPET/PETIS and X-ray CT technique used in this study will be useful in elucidating the mechanisms implemented by plants to adapt not only to drought conditions but also to other environmental changes.

## Data availability statement

The raw data supporting the conclusions of this article will be made available by the authors, without undue reservation.

## Author contributions

YM and FS conceived and designed the experiments. YM, FS, Y-GY, NS, YNo, KE, YNa, MY, NaK, EY, HT, TY, NoK, ST and YU performed the experiments. ST designed and developed the position adjustment software for PET-CT RSA data. YM and FS analyzed the data. YM and FS wrote the manuscript. All authors contributed to the article and approved the submitted version.
