# Supplementary figures and images for: Rice immediately adapts the dynamics of photosynthates translocation to roots in response to changes in soil water environment
Source: Front Plant Sci. 2023 Jan 18;13:1024144. doi: 10.3389/fpls.2022.1024144 (PMC9889367; doi:10.3389/fpls.2022.1024144)

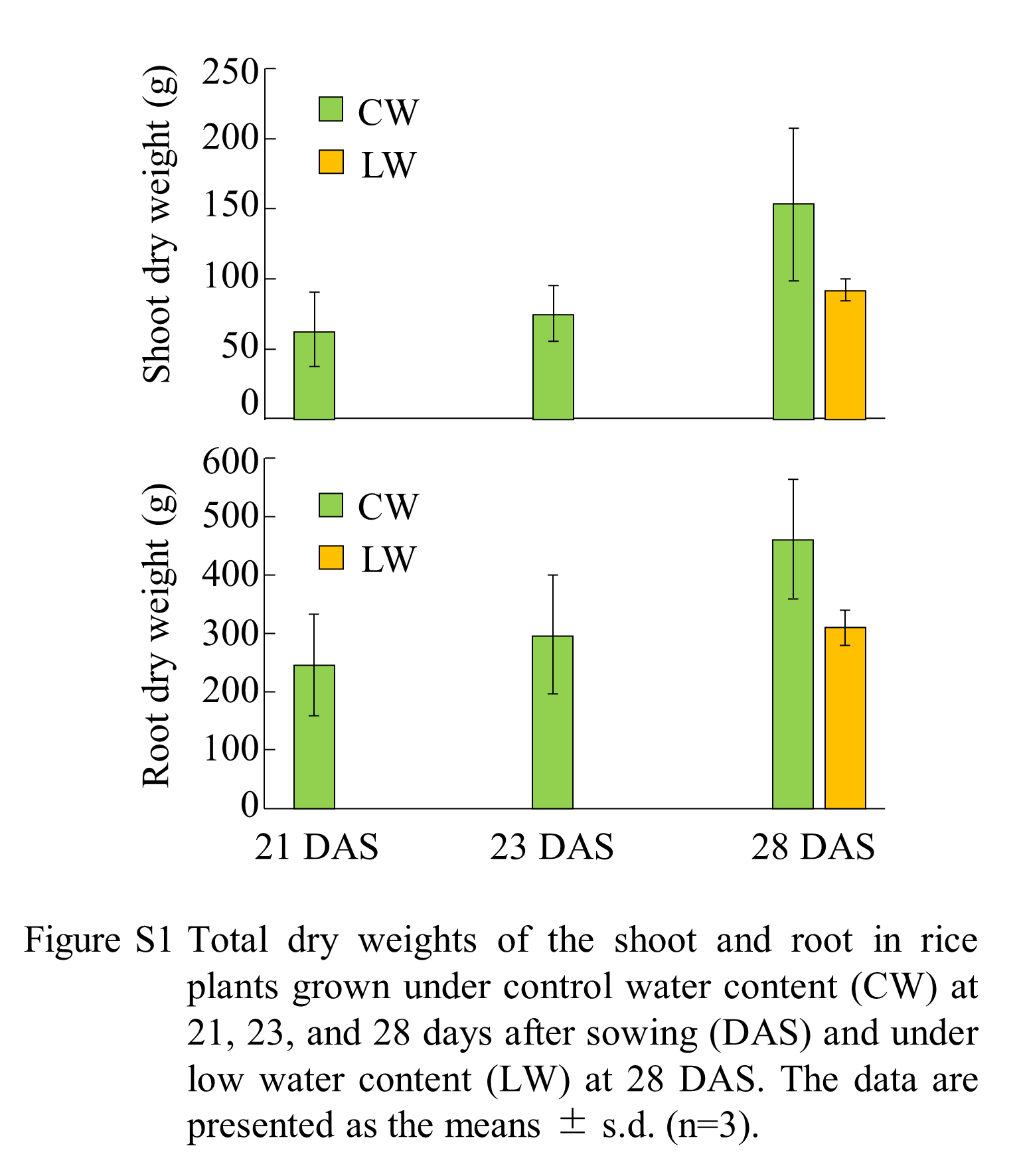

Supplement: Supplementary file 1 [file Image_1.tif]

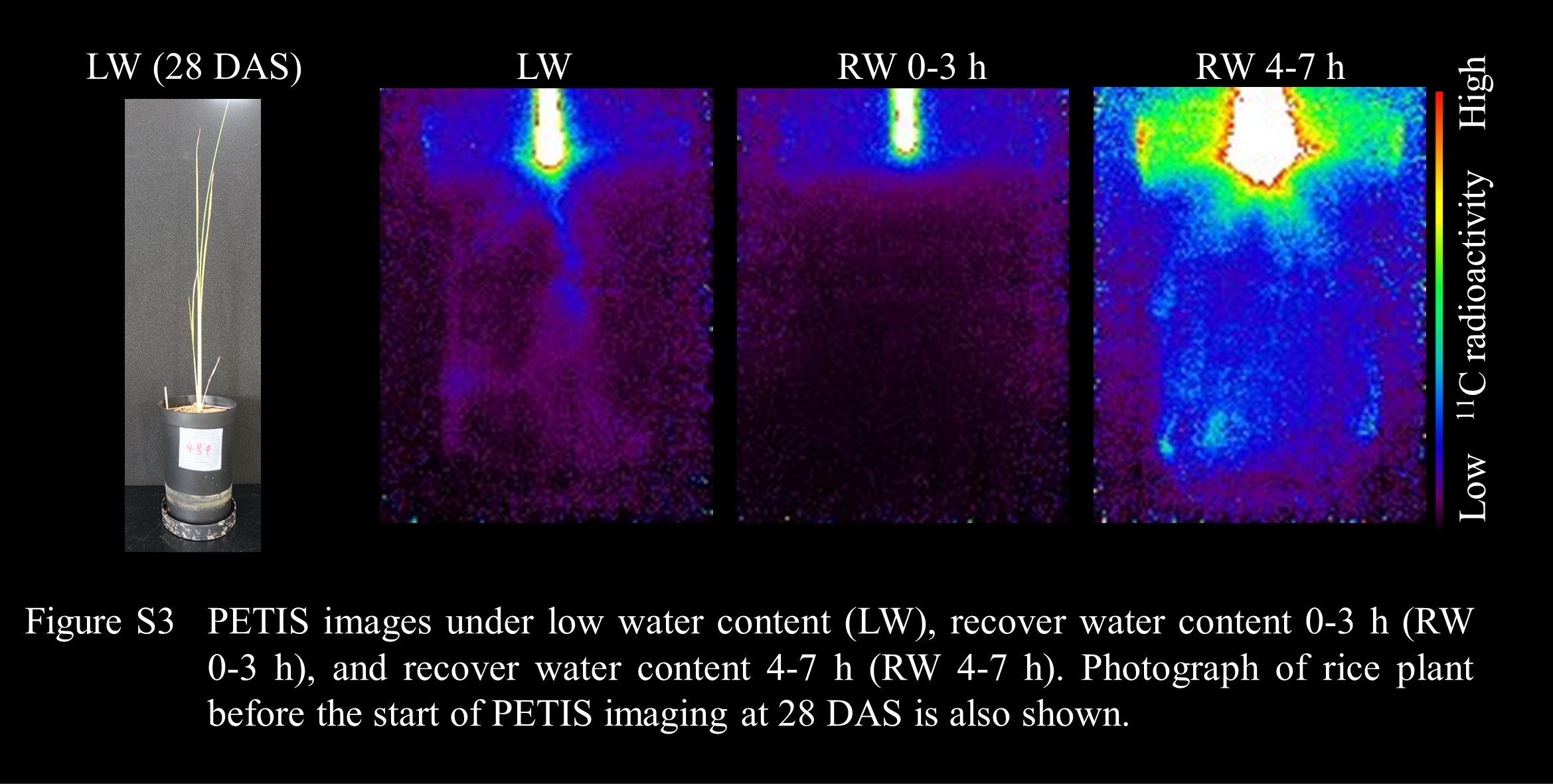

Supplement: Supplementary file 2 [file Image_2.tif]
